# Retained Copper Intrauterine Device Fragment in Pregnancy: A Case Report

**DOI:** 10.7759/cureus.32537

**Published:** 2022-12-14

**Authors:** Justin Chin, Christine M Lomiguen, Haydee Hernandez, Amy Kane

**Affiliations:** 1 Family Medicine, LifeLong Medical Care, Richmond, USA; 2 Medical Education, Lake Erie College of Osteopathic Medicine, Erie, USA; 3 Family Medicine, LECOM (Lake Erie College of Osteopathic Medicine) Health Millcreek Community Hospital, Erie, USA; 4 Pathology, Lake Erie College of Osteopathic Medicine, Erie, USA; 5 Family Medicine, Siouxland Medical Education Foundation, Sioux City, USA; 6 Obstetrics and Gynecology, LifeLong Medical Care, Richmond, USA

**Keywords:** iud fragment, copper iud, pregnancy, covid-19, fragment, copper, copper intrauterine device, copper intrauterine contraceptive devices, paragard, iud

## Abstract

Copper intrauterine device (IUD) failure and fragmentation are rare, with minimal documentation on their effects in pregnancy. Recommendations from professional organizations highlight the importance of prompt identification and surgical removal, as case reports have noted various acute and chronic intra-abdominal pathologies. However, limited guidance exists around counseling patients who are pregnant with a retained IUD fragment. Here, we present a case of a normal pregnancy with a retained copper IUD fragment, while reviewing existing data on management and counseling.

## Introduction

Intrauterine devices (IUDs) are long-acting reversible contraceptives with greater than 99% efficacy. IUDs are divided into hormonal and non-hormonal options, with patient preference, desired symptom control, and associated comorbidities as deciding factors for selection [1]. While there are multiple name brands of the hormonal levonorgestrel-containing IUD, there is only one non-hormonal IUD option in the United States. Paragard® (CooperSurgical, Inc., Trumbull, Connecticut) is a copper-containing IUD in which the metal acts to increase levels of copper ions, prostaglandins, and white blood cells within the uterine and tubal fluids to act as a spermicide to prevent fertilization [2]. Placement and removal require an office visit and can remain for up to 12 years before replacement is needed. Complications from copper IUDs have been reported extensively, particularly related to the migration of complete IUDs or fragments in the setting of continued use after the replacement date [3]. Limited research exists on retained copper IUD fragments and their management options as well as pregnancy outcomes.

Here, we present a case of a retained copper IUD fragment during an otherwise uncomplicated pregnancy along with a review of the literature regarding known complications and outcomes.

## Case presentation

**Figure 1:**
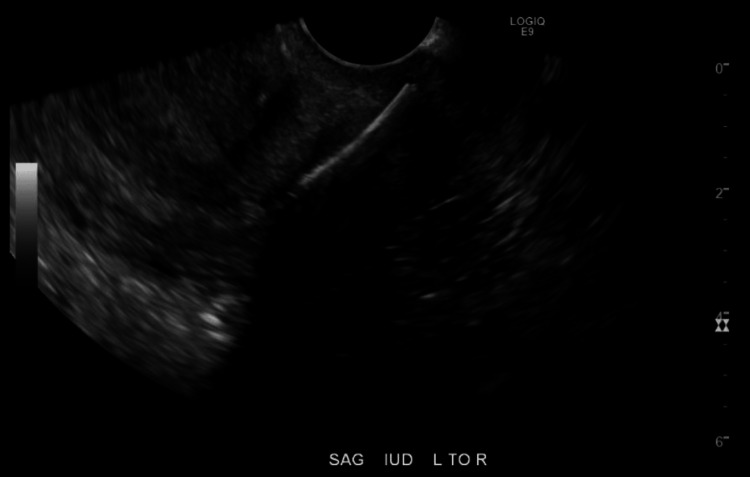
Endovaginal ultrasound in sagittal view with IUD fragment visualized in cervix with posterior acoustic shadowing. SAG: sagittal view; IUD: intrauterine device; L to R: left to right.

Ms. A was a 26-year-old gravida 2 para 1 (G2P1) Yemeni Arabic-speaking woman who presented via telehealth to the obstetrics and gynecology clinic at seven weeks and one day for consultation regarding pregnancy prognosis of the retained arm of a copper IUD status post attempted removal. Her gynecological history was largely unremarkable with menarche at age 15 and no history of sexually transmitted infections. Her obstetric history was positive for one prior term vaginal delivery of a healthy appropriate for gestation age infant at age 24 without any complications. She underwent uncomplicated copper IUD insertion at six weeks postpartum. The IUD remained in place for five years without issue. Her past medical, surgical, family, and social history was non-contributory; however, she frequently declined to use professional interpreters and requested her spouse provide translation during visits. In the preceding week, she had a positive urine pregnancy test with confirmed intrauterine gestation noted on transabdominal and endovaginal ultrasound. Ultrasound findings also noted that her IUD was found in the cervix (Figure 1). During her initial prenatal visit at a family medicine clinic, the risks and benefits of IUD removal were discussed at length and the patient elected for removal. She was prepared in the standard lithotomy position and IUD strings were identified on the speculum exam. Gentle traction with ring forceps was introduced; however, upon inspection of the IUD after removal, one of the arms was not present.

**Figure 2:**
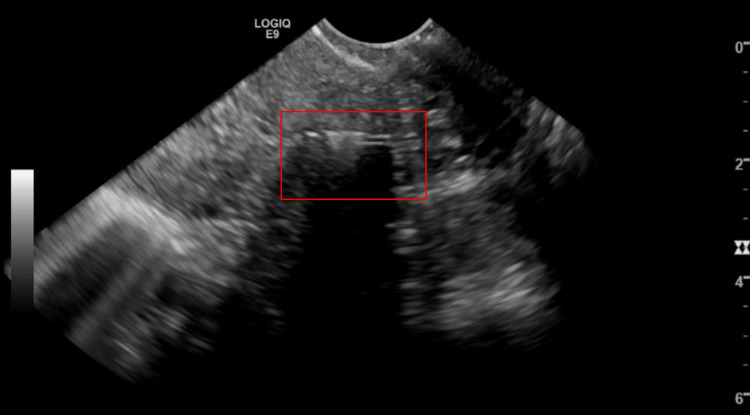
Intrauterine device fragment redemonstrated on endovaginal ultrasound in sagittal view, with a red box highlighting retained fragment.

The patient and her spouse were counseled regarding the limited research regarding retained IUD fragments and outcomes in pregnancy. Recommendations for watchful waiting with close observation and assessment of the location of the IUD fragment at each ultrasound and assessment for fragment at the time of delivery were given. She returned to her family medicine clinic for routine prenatal care with repeat ultrasound at seven weeks and three days demonstrating retained IUD fragment in the lower uterine segment and proximal endocervical canal, with significant distance from the decidual reaction (Figure 2). Her first-trimester labs were unremarkable. Her pregnancy was complicated by the coronavirus disease 2019 (COVID-19) infection at 18 weeks. Subsequent prenatal care was fragmented and she was lost to follow-up after her 20-week ultrasound, which showed normal growth and no IUD fragment. She did not complete her second or third-trimester labs, but briefly re-engaged with care at 38 weeks and two days, in which RhoGAM was given due to O-negative blood type. Point of care ultrasound showed the breech presentation and she was sent to labor and delivery for assessment due to insufficient prenatal care.

While in the labor and delivery unit, she had a normal biophysical profile and non-stress test. The external cephalic version was performed and was successful. Normal spontaneous vaginal delivery of a healthy normal weight infant occurred at 40 weeks and two days. Upon delivery of the placenta, the IUD arm with remaining copper was found embedded in the placental membranes. The patient elected for copper IUD postpartum and underwent uncomplicated insertion at six weeks postpartum.

## Discussion

**Figure 3:**
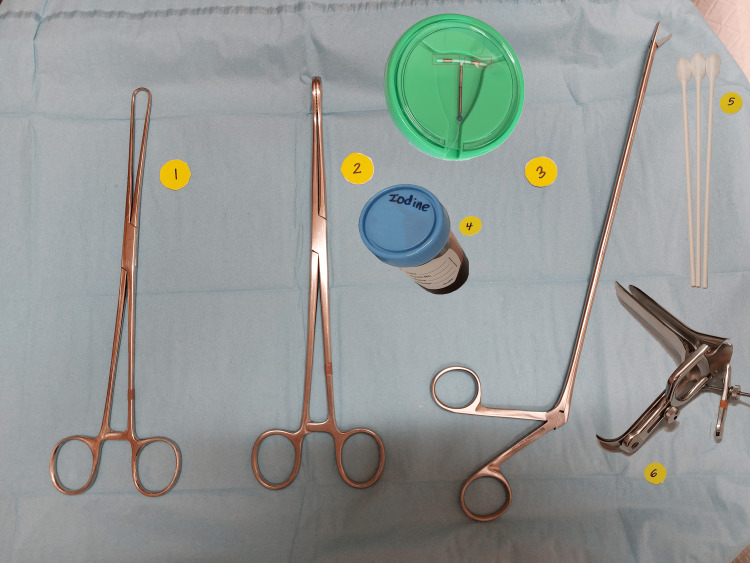
Standard setup for intrauterine device (IUD) removal with uterus model containing a copper IUD. Of note, the arms of the IUD (in red boxes) are the most likely part to be fragmented. The photo was taken by Carmela Asig, MA.

Unintended pregnancy due to failure of copper IUD as a contraceptive measure is rare, with a reported incidence of 1.4 per 100 placements at seven years [4]. In the event of pregnancy with an IUD in place, the American College of Obstetricians and Gynecologists (ACOG) recommends timely removal of the IUD if the strings are visible or if present in the cervix [5-7]. IUD retention in pregnancy has been associated with higher rates of miscarriage and adverse pregnancy outcomes for both the mother and neonate. Term pregnancy with vaginal delivery has been noted with retained IUDs; however, more frequent prenatal care is required due to the increased likelihood of preterm labor and premature rupture of membranes [8]. Once confirmed and if the patient would like to proceed with the pregnancy, IUD removal is often routinely performed in the outpatient clinic with typically no complications to the pregnancy. Upon removal, visual inspection of the IUD is crucial to identify that all parts of the IUD have been removed (Figure 3). With the case patient, she elected for outpatient removal; however, this was complicated by fragmentation. IUD fragmentation or incomplete IUD removal is uncommon, with reported population studies revealing incidence rates of 1.25% and 0.03% among copper and hormone-release IUDs, respectively [9].

Identification of IUD fragments has been documented across all imaging modalities, with ultrasound and magnetic resonance imaging used in pregnancy and computed tomography in non-pregnant patients [10]. The World Health Organization (WHO) has released guidance for prompt surgical intervention upon IUD fragment discovery, with hysteroscopic or laparoscopic removal based on the final location [11]. This does not extend to pregnancy, however, as intra-abdominal and intrapelvic instrumentation are avoided unless there is a life-threatening emergency as the risks outweigh the benefits in fragment removal [12]. Studies have shown increased rates of spontaneous abortion (48%-77%) and preterm delivery (7%-25%) with a retained IUD, along with preterm premature rupture of membranes, chorioamnionitis, septic abortion, and placental abruption [8,13]. There are no studies that describe the prenatal course with IUD fragments, and with this paucity of research along with the stability of the fragment noted on serial ultrasounds, the patient elected to continue the pregnancy without removal.

The literature surrounding management and outcomes for IUD fragmentation is varied, with minimal documentation on its effects in pregnancy. The predominance of research can be found in case reports, which largely attribute complete or IUD fragment migration to findings and symptoms such as abdominal pain, adhesions, and uterine perforation [14-16]. There are no reports of outcomes in relation to retained fragments in pregnancy, likely attributable to the ease of removal once pregnancy is confirmed in the rare case of failure.

For copper and hormonal IUDs, comparable rates have been seen with failure and fragmentation; however, copper IUDs have greater incidence in case reports for migration. This has been hypothesized to be related to the mechanism of action for copper IUDs in which the copper is degraded to release copper ions, which are toxic to sperm. Minimal research exists on copper levels and subsequent pregnancy outcomes; however, the majority of research tends to highlight the consequences of copper deficiency in prenatal care [17]. As seen in this case, natural spontaneous delivery can occur with a retained copper IUD fragment without apparent sequelae. Future studies are needed to determine if migration of fragments can occur in utero as well as further investigate the risks and benefits of fragment retrieval/removal during pregnancy.

## Conclusions

Copper and hormonal IUDs remain a low-cost and safe method for long-acting, reversible contraception despite potential sequelae associated with fragmentation and migration. Failure and fragmentation are uncommon and current recommendations indicate prompt surgical removal. While limited data exist to determine the safety of retained copper fragments in pregnancy, this case reveals the possibility of normal delivery. Greater research is needed to understand outcomes with IUD fragmentation and whether additional counseling is required during birth control selection.
